# Potential Contribution of Aromatase Inhibition to the Effects of Nicotine and Related Compounds on the Brain

**DOI:** 10.3389/fphar.2012.00185

**Published:** 2012-11-06

**Authors:** Anat Biegon, Nelly Alia-Klein, Joanna S. Fowler

**Affiliations:** ^1^Brookhaven National LaboratoryUpton, NY, USA

**Keywords:** smoking, sex, CYP19, extragonadal estrogen, amygdala, PET imaging, vorozole

## Abstract

Cigarette smoking continues to be a major public health problem, and while smoking rates in men have shown some decrease over the last few decades, smoking rates among girls and young women are increasing. Practically all of the important aspects of cigarette smoking and many effects of nicotine are sexually dimorphic (reviewed by Pogun and Yararbas, 2009). Women become addicted more easily than men, while finding it harder to quit. Nicotine replacement appears to be less effective in women. This may be linked to the observation that women are more sensitive than men to non-nicotine cues or ingredients in cigarettes. The reasons for these sex differences are mostly unknown. Several lines of evidence suggest that many of the reported sex differences related to cigarette smoking may stem from the inhibitory effects of nicotine and other tobacco alkaloids on estrogen synthesis via the enzyme aromatase (cyp19a gene product). Aromatase is the last enzyme in estrogen biosynthesis, catalyzing the conversion of androgens to estrogens. This review provides a summary of experimental evidence supporting brain aromatase as a potential mediator and/or modulator of nicotine actions in the brain, contributing to sex differences in smoking behavior. Additional research on the interaction between tobacco smoke, nicotine, and aromatase may help devise new, sex specific methods for prevention and treatment of smoking addiction.

## Introduction

Cigarette smoke and nicotine produce diverse behavioral and physiological effects in the developing as well as the adult human brain (Benowitz, 2008), including changes in cognition, anxiety, and aggression. These effects are traditionally explained by an interaction with nicotinic acetylcholine receptors (nAChR). However, some well established peripheral effects of smoking in women are not easily explained by this mechanism. Specifically, female cigarette smokers reach menopause at an earlier age and have lower plasma estrogen levels than non-smoking females (Daniell, 1972; MacMahon et al., 1982; Nusbaum et al., 2000). Female smokers are also at increased risk of osteoporosis, which is a well known correlate of decreased peripheral estrogen levels (Daniell, 1972; Pant and Shapiro, 2008; Korkor et al., 2009).

## Aromatase

**Figure 1:**
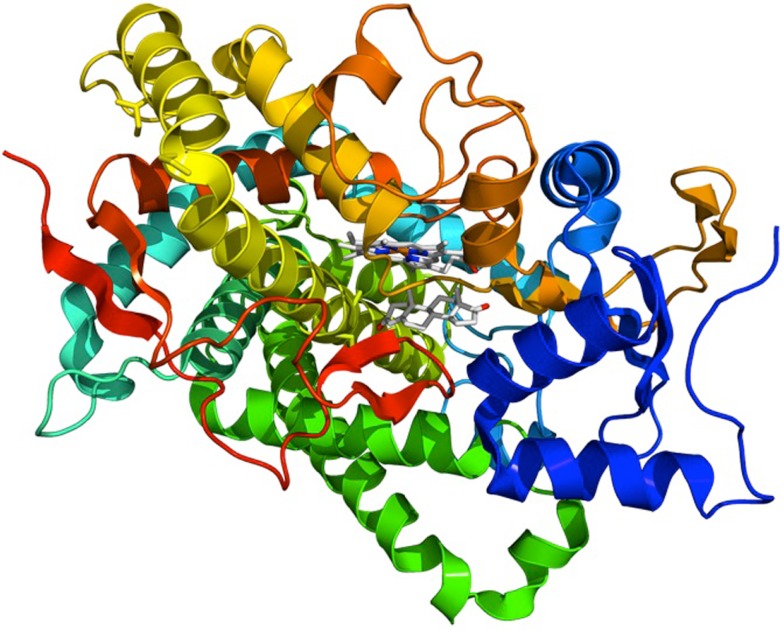
**Structure of aromatse**. Crystallographic structure of the human aromatase cytochrome P450 (rainbow colored cartoon, N-terminus = blue, C-terminus = red) in complex with the cofactor protoporphyrin IX (top) and the substrate androstenedione (bottom) depicted as stick diagrams (carbon = white, oxygen = red, nitrogen = blue, iron = orange; from Wikipedia).

**Figure 2:**
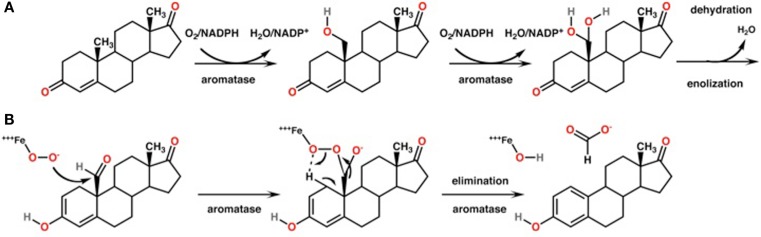
**Biosynthesis of estrgoens by aromatase**. **(A)** General reaction for the conversion of testosterone to estradiol catalyzed by aromatase. **(B)** Catalytic mechanism of aromatase. The methyl group is a oxidized and subsequently eliminated.

Estrogen biosynthesis depends on the enzyme aromatase (Cyp19a gene product, Figure 1), which irreversibly converts androgens such as androstenedione and testosterone synthesized in both the ovary and testes, to the estrogens estrone and estradiol, respectively. The mechanism is depicted in Figure 2. In humans, the gene is located on chromosome 15q21.1 and is composed of 10 exons. Among them, exons II–X encode the aromatase protein and the 3′-untranslated region of the mRNA, whereas the tissue-specific first exon is alternatively spliced giving rise to a differential 5′-untranslated region of the mRNA in different tissues. Correspondingly, tissue-specific promoters are used for tissue-specific regulation of the *CYP19* gene expression (e.g., Kamat et al., 2002). Aromatase has a molecular weight of 55 kDa, a *K*_m_ for testosterone ranging from ∼60 to ∼240 nmol and a *K*_m_ for androstenedione ranging from 0.1 to 30 μM, depending on the source of the enzyme and the lab performing the assay (e.g., Nakajin et al., 1986; Guet et al., 1999; Cooke, 2002; Hong et al., 2007).

## Nicotine and Related Alkaloids Inhibit Aromatase *in vitro*

To explore the possible link between cigarette smoking and decreased endogenous estrogens, Barbieri et al. (1986) examined the effects of constituents of tobacco on estrogen production in human choriocarcinoma cells and term placental microsomes. In choriocarcinoma cell cultures, nicotine, cotinine (a major metabolite of nicotine), and anabasine (a minor tobacco constituent) all inhibited androstenedione conversion to estrogen in a dose-dependent fashion at concentrations in the low micromolar range. Removal of nicotine, cotinine, and anabasine from the culture medium resulted in the complete reversal of the inhibition of aromatase. Furthermore, a supraphysiologic concentration of androstenedione (73 μM) in the culture medium blocked the inhibition of aromatase caused by nicotine, cotinine, and anabasine. In preparations of term placental microsomes, nicotine, cotinine, and anabasine also inhibited the conversion of testosterone to estrogen. Kinetic analysis demonstrated the inhibition to be competitive with respect to the substrate. These findings suggest that tobacco alkaloids exert a direct, competitive, and reversible inhibitory effect on aromatase activity at micromolar concentrations (Barbieri et al., 1986). Importantly, subsequent studies by another research group discovered additional tobacco constituents and nicotine derivatives with a significantly higher (sub-micromolar concentrations) activity in peripheral tissues including human placenta, similar to that of clinically useful aromatase inhibitors (Osawa et al., 1990; Bullion et al., 1991; Kadohama et al., 1993). To date there have been no published studies of the concentrations of nicotine or its analogs required for *in vitro* inhibition of aromatase in brain cells including astrocytes, microglia, and neurons.

The objective of this review is to summarize recent findings documenting the distribution of aromatase in the brain and its inhibition by nicotine *in vivo*; and to examine the implications these findings may have on our understanding of developmental and acute effects of nicotine on brain physiology and behavior. It is important to note that aromatase inhibition is expected to be mostly similar but not identical to estrogen receptor blockade, since inhibition of aromatase not only lowers the absolute levels of estrogens but also may increase testosterone and thus further reduce the estrogen/testosterone ratio in males and females. Due to sex differences in regional morphology, levels of estrogen, testosterone, and their receptors in the brain (e.g., Cahill, 2005), manipulation of aromatase activity is likely to have sexually dimorphic functional outcomes. Therefore, this review is focused on findings in smoking and in aromatase and aromatase inhibitors rather than the vast body of literature related to effects of estrogen or testosterone on the brain, recently reviewed by McCall and Singer (2012) and by McEwen et al. (2012).

## Aromatase is Expressed in the Adult Brain and Inhibited by Nicotine *in vivo*

### Brain expression of aromatase

Aromatase expression is found in many brain regions in birds, rodents, non-human primates, and humans (Roselli et al., 1998; Roselli and Resko, 2001; Biegon et al., 2010b; recently reviewed in Biegon et al., 2010b, 2012; Azcoitia et al., 2011). In rodents, the highest concentration of aromatase were found in amygdala and the bed nucleus of the stria terminalis (e.g., Takahashi et al., 2006) with low though significant levels in other regions (e.g., Sierra et al., 2003).

Non-invasive assessment of aromatase availability throughout the primate brain has become possible following radiosynthesis, initial primate studies (Lidstrom et al., 1998; Kim et al., 2009; Biegon et al., 2010a), and validation in humans (Biegon et al., 2010b) of a radiolabeled aromatase inhibitor suitable for positron emission tomography (PET). PET studies in both rhesus monkeys and baboons revealed that the highest levels of aromatase were found in the amygdala and preoptic area while the thalamus and medulla contained low levels (Lidstrom et al., 1998; Roselli and Resko, 2001; Takahashi et al., 2006; Kim et al., 2009).

**Figure 3:**
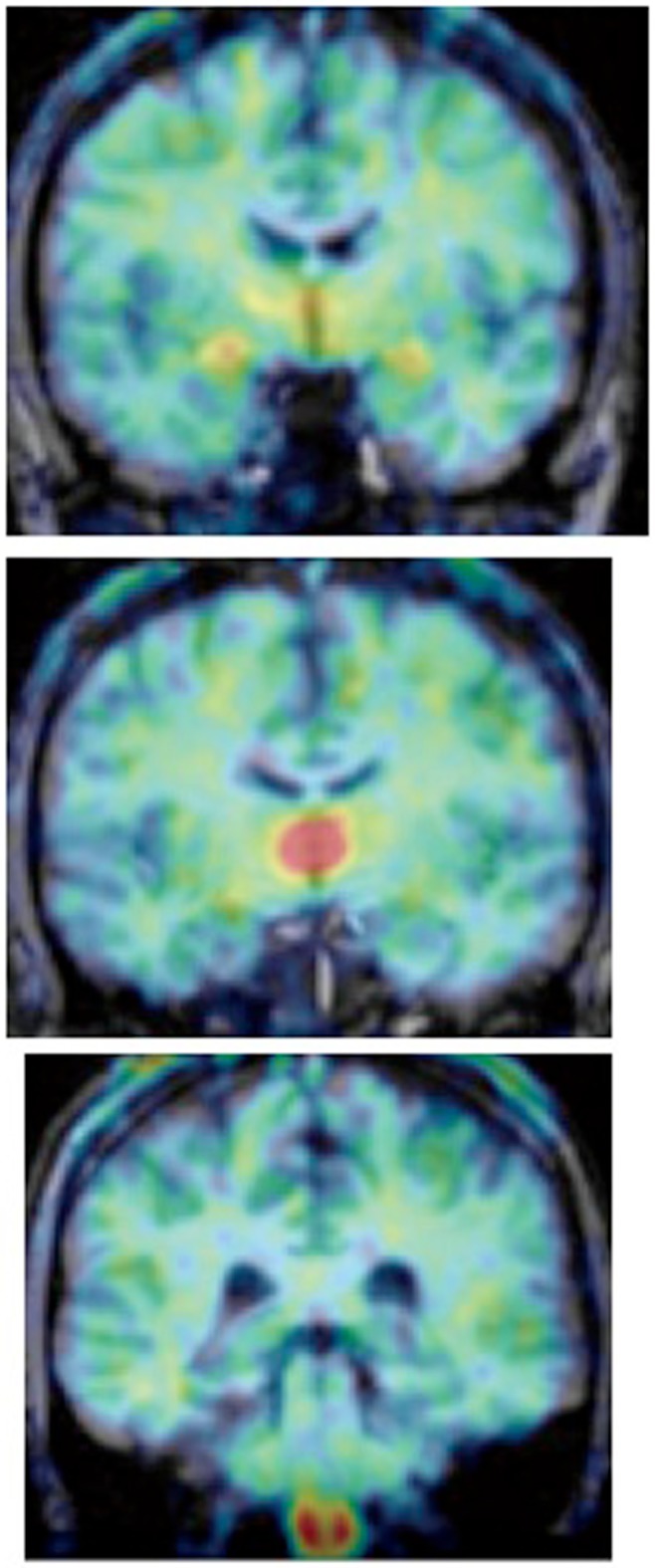
**Regional distribution of aromatase in the living human brain**. Summed frames obtained from PET acquisitions over 90 min after [^11^C]vorozole administration of a representative non-smoking subject were pseudocolored using the rainbow spectrum, such that red regions correspond with high density of radioactivity and blue corresponds to the lowest densities. The PET images are overlaid on a gray-level structural (T1 weighted) MRI scan of the same subject. From left to right: coronal slice at the level of amygdala and hypothalalmus; slice at the level of the medial thalamus and; slice at the level of the medulla (inferior olive; from Biegon et al., 2010b).

The most comprehensive study of aromatase distribution in the human brain to date, performed using PET and [^11^C]vorozole (Biegon et al., 2010b), revealed a highly specific and heterogeneous pattern which appears to be unique to humans. The highest levels were seen in the thalamus, though thalamic distribution was not uniform: within the thalamus, the highest levels were found in the dorsomedial and pulvinar nuclei with lower density in reticular, lateral, and anterior-ventral thalamic nuclei (Figure 3). Very high levels were also found in the paraventricular hypothalamic nucleus.

Moderately high levels of aromatase were noted in amygdala and preoptic area/anterior hypothalamus and in the medulla (inferior olive). Basal ganglia levels were relatively low, with visibly higher levels in the ventral striatum/nucleus accumbens (Biegon et al., 2010b). Labeled vorozole distributed to all cortical regions, with hippocampus indistinguishable from the temporal cortex. The distribution volume values derived from a two compartment model (Gunn et al., 2001; Logan, 2003; Logan et al., 2011) in both men and women (regardless of menstrual cycle) followed the rank order: thalamus > amygdala = preoptic area > medulla (inferior olive) > cortex = hippocampus, putamen, cerebellum, and white matter.

Earlier published postmortem studies were limited to a small number of preselected regions, although the combination of studies by different research groups and different methodologies support the notion that aromatase is ubiquitous in the human brain, although only a few regions express high levels of the enzyme (reviewed in Azcoitia et al., 2011 and Biegon et al., 2012).

To elaborate, aromatase *gene expression* was examined in postmortem samples from eight brain regions (Sasano et al., 1998). The amount of aromatase mRNA determined by RT-PCR assay in six cases (four men, two women) was highest in pons, thalamus, hypothalamus, and hippocampus. Analysis of multiple exons 1 revealed that exons 1f, considered specific for brain, as well as 1b (fibroblast type) and 1d (gonadal type), were expressed in the brain. Exons 1d and 1f tended to be utilized in hypothalamus, thalamus, and amygdala. The amount of overall mRNA expression was also higher in hypothalamus, thalamus, and amygdala than in other regions of the brain. There were no differences of utilization of exons 1 and mRNA expression of aromatase between female and male brain. The authors concluded that their results demonstrate that aromatase is expressed widely in human brain tissues in both men and women. The presence of aromatase transcripts in human temporal cortex, frontal cortex, and hippocampus was also confirmed by Stoffel-Wagner et al. (1999).

Aromatase *immunoreactivity* was found in hypothalamus, amygdala, preoptic area, and (cholinergic) ventral forebrain nuclei (Ishunina et al., 2005) in humans. Additional studies confirmed aromatase immunoreactivity in temporal cortex, hippocampus, and prefrontal cortex (Yague et al., 2006, 2010). Immunohistochemistry was also used to examine the cellular and subcellular distribution of aromatase in the human brain, establishing the presence of aromatase immunoreactivity in neurons as well as in glia. Thus, cortical and hippocampal aromatase was detected in pyramidal cells, granule cells, and interneurons; in perikarya, dendrites, axons, and axon terminals (Naftolin et al., 1996; Yague et al., 2006, 2010). The presence of glial aromatase was confirmed in prefrontal cortex, temporal cortex, and hippocampus, where it was associated with astrocytes (Yague et al., 2006, 2010).

Aromatase enzymatic activity was first described in the fetal human limbic system by Naftolin et al. (1971), followed by reports on activity in the adult brain and temporal cortex (Naftolin et al., 1996; Steckelbroeck et al., 1999). In agreement with the results from other methods described above, there were no differences in aromatase enzymatic activity between men and women and no significant effect of aging in these brain regions (Steckelbroeck et al., 1999).

### Nicotine administered *in vivo* inhibits aromatase in the brain

Nicotine modulation of brain aromatase *in vivo* was first reported by von Ziegler et al. (1991) in fetal and neonatal mice. After 1 or 2 weeks of prenatal exposure to 6 mg/kg nicotine delivered by an osmotic minipump, aromatase activity in male forebrains was significantly decreased at postnatal day 6, with no significant effects in females. The authors initially reported that nicotine alters brain aromatase activity only on postnatal day 6, the day when normal females show lower levels than normal males (von Ziegler et al., 1991). In subsequent studies in rats, it was shown that both nicotine and cotinine inhibited aromatase activity in the basal forebrain of male fetuses. Nicotine was twice as effective as cotinine and the effects of the two drugs were additive (Sarasin et al., 2003).

**Figure 4:**
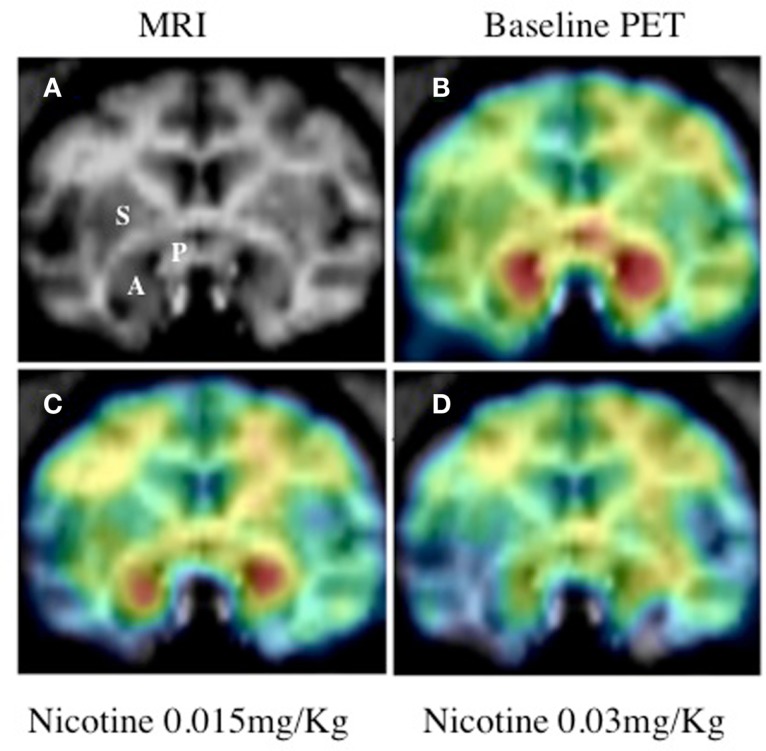
**Effect of nicotine on brain aromatase availability in the female baboon**. Top: **(A)** Baboon brain MRI, coronal section at the level of amygdala. S, striatum; P, preoptic area; A, amygdala. **(B)** Representative baseline PET image coregistered with MRI. **(C)** PET image of same baboon following low dose nicotine (0.015 mg/kg). **(D)** PET image following injection of high dose (0.03 mg/kg) nicotine. PET images show averaged frames acquired between 52.5 and 90 min after tracer injection, pseudocolored using the rainbow spectrum, with purple indicating the lowest density and red indicating the highest density of radioactivity (from Biegon et al., 2010a).

Using [^11^C]vorozole, we have recently shown that acute *in vivo* exposure to nicotine doses which produced plasma levels similar to those found in smokers, resulted in significant region- and dose-dependent decreases in aromatase availability in the female baboon brain. The largest and most significant inhibition was found in the amygdala, where intravenous injection of 0.03 mg/kg nicotine reduced brain aromatase availability by ∼50% (Biegon et al., 2010b; Figure 4).

## Brain Effects of Nicotine: Similarities and Differences with Aromatase Inhibition

The discovery of the ability of neuroendocrine tissues to aromatize androgens to estrogens was crucial to the formulation of the aromatization hypothesis (Naftolin and Ryan, 1975; McEwen et al., 1977), stating that testosterone synthesized by the fetal testis diffuses into the male brain where it is locally aromatized to estradiol and then initiates the process of masculinization, resulting, in adults, in the capacity to express male-typical sexual behaviors and high levels of aggression. In females, the aromatization process affects such functions as mood and appetite.

Consequently, aromatase inhibition can have a variety of sexually dimorphic effects on multiple domains including sexual functioning, mood, and cognition but the nature and persistence of the effects can vary greatly depending on the developmental status during exposure. The sections below provide a review of the brain effects of nicotine and aromatase inhibition. We highlight developmental, “organizational” brain effects (i.e., effects of maternal smoking during pregnancy) as well as effects of exposure in adolescents and adults when available in the literature, with the best supported similarities and differences summarized in Table 1.

**Table 1:** **Similarities and differences in brain effects of nicotine and aromatase inhibition**.

	Male		Female	
	Nicotine	AI	Nicotine	AI
Sexual behavior			
Prenatal exposure	Decrease	Decrease	Increase	Increase
Adult exposure	Decrease	?	Decrease	Decrease
Anxiety/Depression			
Prenatal exposure	No effect	No effect	Increase	Increase
Adult exposure	No effect	No effect	Decrease	Increase
Hot flashes			Increase	Increase
Weight gain	Decrease	Decrease	Decrease	Increase

*AI, aromatase inhibition. Nicotine refers to smoking as well as nicotine alone*.

*?, there is not enough data on males to justify an increase or decrease effect*.

### Sexual behavior

Prenatal nicotine exposure was shown to decrease male sexual behavior and to demasculinize male offspring (Segarra and Strand, 1989), paralleling the effects in aromatase “knock-out” (ArKO), and prenatal treatment with AI. In adult males, acute nicotine administration resulted in decreased intromission frequency, though this occurred only at 1.6 mg/kg, the highest dose tested (Retana-Marquez et al., 1993). Decreased sexual performance was also self reported in men exposed to cigarette smoke (Weisberg, 1985).

Studies (reviewed in Roselli et al., 2009) confirmed that male copulation is severely impaired in ArKO domestic mice in which the aromatase gene was selectively inactivated, consistent with the role for aromatization in both the organizational and activational effects of testosterone. Testosterone administration did not improve male sexual behavior in castrated ArKO adults, whereas combined treatment with estradiol and dihydrotestosterone (a non-aromatizable androgen) almost completely restored copulation behavior to levels observed in wild-type males. These results suggest that estrogens derived from aromatization of testosterone exert major activation effects on coital behavior in male C57Bl6 mice. The extent to which testosterone in men acts through aromatization to estradiol, is not yet clear. In eugonadal men, the estrogen receptor antagonist, tamoxifen, and the aromatase inhibitor, testolactone, had no adverse sexual effects. Furthermore, dihydrotestosterone was as effective as testosterone in maintaining sexuality in hypogonadal men, suggesting that aromatase was not involved (Gooren, 1985). The comparison of two men with congenital aromatase deficiencies, one with accompanying hypogonadism, suggested that testosterone alone allows for a normal sexual activity, but that there is a synergistic effect between testosterone and estradiol derived from aromatization. These findings suggest that aromatization may be required in men for completely normal sexual behavior, but that androgens are the main steroids involved.

In human females, prenatal exposure to nicotine resulted in a significant increase in same-sex orientation among female offspring (Ellis and Cole-Harding, 2001). The influence of aromatization on female sexual behavior is less clear but studies in female rats show that sexual behavior was enhanced by prenatal inhibition of androgen aromatization (Clemens and Gladue, 1978). In humans, gender role and sexual behavior are disrupted in girls with congenital adrenocortical hyperplasia, which increases testosterone levels (see recent review by Barenbaum and Beltz, 2011). Acute effects show that treatment in adult female rats increased lordosis in estrogen-treated ovariectomized female rats (Fuxe et al., 1977; Weaver and Clemens, 1987), but acute exposure to nicotine in adult men and women significantly reduced sexual arousal (Harte and Meston, 2008a,b). In addition, adult women given aromatase inhibitors report a loss of libido (Mitwally and Casper, 2003; Zivian and Salgado, 2008). These few studies on the sexual behavior of females may not be sufficient to establish a solid hypothesis on the direction of effects.

### Aggression

Prenatal smoking exposure and high trait aggression are associated variables in numerous animal and human studies, particularly during adolescence (Escobedo et al., 1997). This effect has been replicated in epidemiological studies and after controlling for important variables of heritability as mother’s antisocial behaviors (Moffitt et al., 2008; Wakschlag et al., 2010). The observation that tobacco smoke exposure inhibits brain monoamine oxidase A (MAO A; Fowler et al., 1996), has led to the suggestion that the mechanism responsible for aggressive behavior in the offspring of females exposed to cigarette smoke during pregnancy is MAO A inhibition (Wakschlag et al., 2010). Indeed brain MAO A activity predicts trait aggression at adulthood (Alia-Klein et al., 2008, 2009). An additional and perhaps interactive mechanism in the effects of smoking on aggression is the inhibition of aromatase and little is known about the interactive potential of chronic MAO and aromatase inhibition.

Prenatal and developmental effects of smoking on aggression appear to be different than the acute effects of smoking in adults. In adults, smoking appears to reduce irritability and aggression. Furthermore, nicotine replacement therapy was found useful in reduction of agitation and aggression in smokers with schizophrenia (Allen et al., 2011).

Importantly, although numerous preclinical and clinical studies have shown that acute nicotine treatment reduces aggression, smoking deprivation results in negative mood, aggression, and hostility in adult rodents and humans (Schechter, 1974; Cherek, 1981). In a recent study, a lifetime history of cigarette smoking was associated with high traits of aggression and impulsivity in healthy and personality disorder participants and these effects may have started during prenatal development (Dakwar et al., 2011).

The effects on females appear to be similar to males in that smokers of both sexes exhibit negative emotionality as compared to non-smokers; however, the data is suggestive that smoking affects aggression in males more than in females, where it has a primary effect on depression (Pogun and Yararbas, 2009).

Aggressive behavior, long thought to be controlled by testosterone, also appears to be strongly dependent on aromatization and estrogen in both mammalian and non-mammalian species (see review by Trainor et al., 2006). Estrogen has been shown to modulate aggression in a variety of species. Although in most cases estrogen increases the probability and intensity of male-on-male aggressive behavior, there are exceptions in which estrogen decreases the intensity of aggression. Thus, the duration of aggressive behavior in resident-intruder tests was extremely low for ArKO mice compared to wild-type mice and estradiol injections restored aggression to wild-type levels. However, when the production of estrogen was blocked by an AI (fadrozole) in California mice, the males were more aggressive compared to controls, indicating that production of estrogen is associated with reduced aggression in California mice.

### Depression and anxiety

Developmental effects of prenatal smoking exposure increase the risk for depression particularly in females. Female offspring of rodents prenatally treated with nicotine show vulnerability during adolescence to depression and early smoking onset; more so than males (Romero and Chen, 2004; Vaglenova et al., 2004). Findings from human and animal studies infer sex and region specific effects and suggest a role for smoking in higher rates of depression, especially among adolescent females (Kandel et al., 1994; Cornelius and Day, 2009). Smoking during pregnancy has psychological effects on the mothers as well as their offspring. In a study of persistent pregnant smokers, the smokers reported higher prenatal stress and depression than non-persistent smokers or non-smokers (Eiden et al., 2011). Women were also found to be more vulnerable to the depressive symptoms of nicotine withdrawal (Gaffin et al., 2011). Acutely, nicotine has been found to be more anxiolytic in female than in male rats (Harrod et al., 2004), corroborating human studies which also document that females are more susceptible to the effects of nicotine on anxiety (Pogun and Yararbas, 2009).

In males nicotine has been shown to produce antidepressant-like responses in rats subjected to the forced swimming test, a popular model of depression in rodents. While this effect is not expected to reflect inhibition of aromatase, it is relevant to note that orchiectomy abolished the antidepressant effect of nicotine and its restoration required supplementation with estradiol (Bonilla-Jaime et al., 2010).

Decreased aromatase availability appears to have sexually dimorphic effects on depression and anxiety in rodents. ArKO females displayed decreased active behaviors, such as struggling and swimming, and increased passive behaviors, such as floating, in repeated sessions of the forced swim test, indicating that these females exhibit “depressive-like” symptoms (Dalla et al., 2004). This effect was not observed in males (Dalla et al., 2005). By contrast, ArKO males did not differ from WT in spontaneous motor activity, exploration, or anxiety. These findings are in line with the absence of major neurochemical alterations in hypothalamus, prefrontal cortex, or striatum, which are involved in the expression of these behaviors.

Clinical and community studies of women have shown that both anxiety and depression are increased in women taking aromatase inhibitors for breast cancer (Mitwally and Casper, 2003; Zivian and Salgado, 2008). Long-term efficacy and safety of the use of AI in men and boys has not been established to date, although sex differences in brain-related effects are likely due to the inherently different implications of changing the estrogen/androgen ratio in males and females and also because higher levels of testosterone in males compared to females renders it more difficult to effectively inhibit aromatase in men (de Ronde and de Jong, 2011).

### Appetite

Prenatal exposure to nicotine appear to alter saccharin preferences in a sexually dimorphic manner, eliminating an observation in untreated animals where females had a higher preference to saccharin than untreated males. In the animals exposed to nicotine prenatally, there was an increase in males preferring saccharin to the female level (Lichtensteiger and Schlumpf, 1985). This study provides another example of a demasculinizing effect of prenatal nicotine as would be expected to result from prenatal aromatase inhibition.

The situation is different in adults, where acute nicotine administration is considered to be a powerful appetite suppressant. In fact, the effects of smoking on body weight are a concern since more women than men report smoking to avoid weight gain (Pomerleau and Kurth, 1996). This effect is at least partly biologically based since studies of Sprague-Dawley rats showed greater effects of nicotine on food intake and body weight in female than male rats (Grunberg, 1986). The appetite-suppressing effect of nicotine appears to be mediated through direct interaction with neurons involved in initiation of feeding behavior (Mineur et al., 2011) and is not likely to be mediated through aromatase inhibition, though other effects of nicotine related to appetite may rely on this mechanism. Thus, nicotine appears to increase the reinforcing properties of food (reviewed in Donny et al., 2011) and older studies report increased consumption of sweet foods following nicotine deprivation (Hughes et al., 1991; Spring et al., 2003).

Estrogen is known to suppress feeding and weight gain in mammalian females including women. An important contribution to this effect of estrogen is suppression of feeding by direct action with brain centers engaged in appetite control (Nunez et al., 1980; Butera and Beikirch, 1989; Dagnault and Richard, 1997; reviewed in Geary, 2004). Consequently, inhibition of aromatase is expected to increase feeding and weight gain, as reported by Kubatka et al. (2008a,b) who found increased weight gain in female rats treated with two different aromatase inhibitors – anastrozole and letrozole. Increased weight gain was reported by close to 50% of a community sample of 1200 women taking aromatase inhibitors for breast cancer (Zivian and Salgado, 2008). In the same vein, we found a negative correlation between aromatase in the amygdala and BMI, which was more pronounced in women than in men (Wang et al., 2011). However it is noteworthy that loss of appetite is also reported by some women receiving aromatase inhibitors (Mitwally and Casper, 2003) amounting to 8% of responders in a community sample (Zivian and Salgado, 2008). There appear to be important sex differences in the control of food intake (Geary and Lovejoy, 2008). Interestingly, in male rodents, peripubertal inhibition of aromatase leads to a decrease rather than an increase in body growth and weight gain (Bajpai et al., 2010).

### Hot flashes

Current smoking (and high BMI), reportedly predispose women to more severe or frequent hot flashes (Whiteman et al., 2003). In perimenopausal women ages 45–54, current smokers had significantly higher androstenedione levels and a higher androgen to estrogen ratio than never smokers. Current smokers also had lower progesterone levels and increased odds of experiencing hot flushes compared to never smokers (Cochran et al., 2008). This effects is sex specific since there are no reports on hot flushes in healthy male smokers. It is noteworthy that men treated for prostate cancer with androgen ablation therapy (e.g., by injections of gonadotropin-releasing hormone analogs) report daily hot flashes, which respond to estrogen treatment (Gerber et al., 2000).

Hot flashes are the most common adverse effect reported in clinical trials and community studies of aromatase inhibitors in women, with up to 75% of users reporting this adverse effect (Bonneterre et al., 2000; Mitwally and Casper, 2003; Howell et al., 2005; Zivian and Salgado, 2008). Hot flashes arise when the thermoregulatory centers in the hypothalalmus are deprived of estrogen (see review by Rossmanith and Ruebberdt, 2009) and are a classical symptom of estrogen deprivation resulting from menopause and ovariectomy as well as the most common side effect of estrogen receptor antagonists and selective estrogen receptor modulators like tamoxifen (e.g., Bonneterre et al., 2000; Howell et al., 2005).

### Cognition and neuroprotection

Prenatal exposure to nicotine impairs cognitive functions, such as memory and attention, and this impairment is gender-specific. Deleterious effects in male but not female offspring were documented on synaptic function, cell signaling, and cell number. In terms of cognitive performance, marked deficits were observed in males in auditory attention (Niaura et al., 2001; Cornelius and Day, 2009). Other studies show that on average, male smokers perform more poorly than female smokers on attention and memory tasks (Abreu-Villaca et al., 2003; Jacobsen et al., 2007).

In females, stimulation of estrogen receptors, which enhances hippocampal neurogenesis and synaptic plasticity, may be protective through providing greater adaptive capacity (Tanapat et al., 1999; McEwen, 2002). However, this protection relative to males is diminished in prenatally exposed rats who were treated with nicotine later in adolescence (Slotkin et al., 2007). Human studies also documented that nicotine exposed females showed reduced performance accuracy in neuropsychological tasks (Niaura et al., 2001; Cornelius and Day, 2009).

A meta analysis of the acute effects of nicotine and smoking on adult human performance found significant positive effects of nicotine or smoking in several domains, including short-term and working memory, with effect sizes ranging from 0.16 to 0.44 (Heishman et al., 2010). Treatment with nicotine receptor agonists has been shown to elicit improvement of cognitive performance in a variety of behavioral tests in rats, monkeys, and humans (Reviewed in Mudo et al., 2007).

Evidence for the involvement of aromatase in support of cognitive function and neuroprotection in various species was recently reviewed by Garcia-Segura (2008) and Roselli et al. (2009). More recently, AI were found to inhibit hippocampal learning (long-term potentiation, LTP) and dendritic spine formation (Zhou et al., 2010; Vierk et al., 2012) although the effects on LTP appear to be sexually dimorphic, with significant effects found in female but not male mice. In humans, a testosterone-supplementation study in elderly men showed that the addition of an AI reversed the beneficial effects of testosterone on verbal memory with no effect on spatial memory (Cherrier et al., 2005). A similar study in women did not reveal statistically significant effects of AI, although the authors suggest the small size of the study may be a contributing factor (Shah et al., 2006).

In preliminary studies at our laboratory, availability of aromatase in the amygdala was negatively correlated with performance on verbal learning and memory tests in men, while in women, aromatase in the amygdala was positively correlated with high scores on trait constraint (Biegon et al., 2011a,b). Thus, although there were no sex differences in aromatase availability in amygdala or learning and memory scores nor traits in our sample, the amygdala aromatase availability was associated with sex specific effects on verbal learning, memory, and personality traits.

### Alzheimer’s disease

Acute nicotine injections and chronic treatment with nicotine patch have been found to improve attentional performance in patients with Alzheimer’s Disease (AD; White and Levin, 1999). The neural substrate for nicotine-induced improvement in attention is hypothesized to be related to the basal forebrain cholinergic system (Lawrence and Sahakian, 1998).

Epidemiological studies initially indicating a lower incidence of AD in smokers now suggest conflicting results. Clinical and pathology findings also are mixed as to how smoking behavior affects the manifestation of AD markers and the relevance of aromatase inhibition is unclear. Studies that show nicotine-induced increases in nAChR and protection against age-related nAChR decline, contrast perhaps in a functionally relevant way, to losses of nAChR in AD. Although epidemiological, clinicopathological, and functional studies in humans do not present a cohesive picture, much *in vitro* data suggest neuroprotective properties of nicotine when used in models of neurodegenerative disorders. Studies of nicotine and nicotinic agonist effects on cognitive function in the non-demented and in AD are not compelling. More work is needed to ascertain whether acute or repetitive activation of nAChR with acute or intermittent exposure to nicotine or the persistent inactivation of nAChR with chronic nicotine exposure is a therapeutic objective and/or explains any pro-cognitive effects of those drugs. Other studies show complex interactions between nAChR, nicotinic agonists, and agents implicated in AD etiology (reviewed in Sabbagh et al., 2002).

Aromatase has been suggested to play a role in AD (for review see Hiltunen et al., 2006) although available data are not sufficient to establish a neuroprotective effect. Human genetic studies have revealed evidence of a relationship between mutations of the aromatase gene and AD risk. Subjects who had single nucleotide polymorphisms (SNPs) in the CYP19 gene were reported to have an ∼60% increase in the risk for AD (Iivonen et al., 2004). Likewise, a twofold increased risk for AD was observed in ApoE ε4 carriers who had SNPs in the CYP19 gene (Huang and Poduslo, 2006). Studies on postmortem brains revealed enhanced aromatase expression in the nucleus basalis of Meynert and decreased aromatase expression in the hypothalamus of AD patients, with no sex differences observed in any of the nuclei studied (Ishunina et al., 2005). In contrast, in hippocampus samples from women, estrogen receptors (ERα subtype), and aromatase expression were found to increase with age, and to decrease in AD (Ishunina et al., 2007). An earlier study on frontal and temporal cortical samples did not find significant difference in aromatase activity between AD brains and controls (Wozniak et al., 1998).

### Addiction

The rates of cigarette smoking have declined over the last few decades, but smoking decreases among women are less pronounced (Mathers and Loncar, 2006) and smoking rates among girls and young women are increasing (CASA investigators, 2006). Paralleling animal studies, human females appear to become addicted faster than men, while finding it harder to quit (Bohadana et al., 2003). In offspring of women who smoked during pregnancy, the odds of progressing to nicotine dependence were almost doubled compared to offspring from non-smoking women (Cornelius and Day, 2009). Furthermore, nicotine replacement in the context of smoking cessation appears to be less effective in women (Bohadana et al., 2003; CASA investigators, 2006). A question remains how or through what mechanism might cigarette smoking become a gateway to smoking addiction and to other drugs. Another question is how observed sex differences in smoking age of onset and rates of smoking cessation may be related to the actions of aromatase inhibition by smoking.

There is no evidence that aromatase inhibitors are addictive, in stark contrast to the potent addictive properties of nictoine and smoking (Nusbaum et al., 2000; Bohadana et al., 2003). However, several lines of evidence suggest that aromatase may modulate various aspects of nicotine addiction in a sex specific manner. Addiction to other substances is also impacted. Female animals consume more alcohol than males and interestingly, this effect is reversible by gonadectomy (Almeida et al., 1998). This effect is not restricted to alcohol but is also observed with psychostimulants as cocaine.

Sex differences are also found in the context of relapse following periods of abstinence, where as with cigarette smoking, females find it harder to quit than males.

Cigarette smoking is very common among drug addicted individuals, and it appears that methadone, used to facilitate treatment of opioid addiction, is metabolized by aromatase and may act as a potent inhibitor of aromatase *in vivo*. These findings may contribute to variability in methadone clearance, to drug–drug interactions, and to side effects observed in male and female patients. Because methadone seems to be able to inhibit aromatase, it follows that methadone may alter the metabolism and disposition of endogenous testosterone and androstenedione. Lower concentrations of estradiol have been documented in men taking methadone (Hallinan et al., 2009), and low bone mineral density that may be due to low estrogen concentrations has been documented in 83% of patients in a methadone maintenance treatment program (Kim et al., 2006). It is conceivable that other side effects of methadone, which include flushing, muscle pain, and symptoms reminiscent of estrogen withdrawal (Senay, 1985; Backstrom, 1995), may be explained in part by the drug’s action on aromatase.

## Discussion and Conclusion

As shown above, aromatase and its activity in the brain can be parsed to basic psychological processes; effects are observed in affect, learning, and memory, libido, and appetite; and sex differences in the direction and size of the effects are very common. The effects of smoking can also be parsed to the same psychological processes, and sex differences in nicotine action have been recognized in animals as well as human subjects (e.g., recent review by Pogun and Yararbas, 2009).

This literature review highlights findings from animals and humans indicating that smoking and nicotine target brain functions which are also shaped and influenced by aromatase activity. While it has been extensively documented that nicotine interacts with the nicotinic acetylcholine receptor as its mechanism of action, this does not exclude additional mechanisms contributing to the effects of smoking and nicotine on aggression, cognitive function, anxiety, depression, sexual drive, and appetite (e.g., Fowler et al., 1996).

Available findings summarized above highlight the possibility that several of the known effects of nicotine, most prominently effects on sexual and depression-like behavior and induction of hot flashes, may be mediated or modulated by changes in aromatase activity (Table 1) in an age- and sex-dependent manner. It is noteworthy that these effects of nicotine are more prominent in periods such as prenatal development, adolescence and the perimenopausal period, which are associated with relatively abrupt changes in estrogen synthesis (a prenatal peak in brain aromatase in males, a peripubertal increase in ovarian production in girls and a perimenopausal decrease in oarain production in adult women). Such a profile is to be expected from a partial rather than complete inhibition of aromatase activity exerted by nictoine and similar compounds which act as competitive inhibitors of aromatase with low to moderate affinity (Barbieri et al., 1986; Biegon et al., 2010a). Thus, we have shown that nicotine doses producing plasma levels comparable with those found in smokers are capable of a significant but partial (∼50%) inhibition of aromatase. Obviously, more research is indicated to substantiate and better understand the effects of aromatase inhibition on brain function in this context. However, the notion that aromatase inhibition by nicotine and other tobacco alkaloids is an alternative/additional mechanism for the effects of cigarette smoking on human behavior, provides a mechanistic explanation to observed sex differences in smoking and a possible source of new treatment and prevention approaches for the initiation, physiological and psychological consequences, and for the cessation of smoking.

## Future Perspectives

While research on the brain effects and mechanisms of nicotine action has been ongoing for many decades, specific AIs are relatively new agents. Furthermore, despite the fact that several AIs are approved drugs used in the clinical setting, the foremost clinical use of these agents is in the adjuvant treatment of breast cancer (e.g., Buzdar and Howell, 2001), and clinical studies of breast cancer do not include systematic investigation of neuropsychological effects. Consequently, there is a great need for additional basic research on the effects of aromatase inhibition in males and females across the life span. Such studies, as well as side-by-side comparisons of aromatase inhibitors and nicotine and other tobacco constituents have the potential to shed light on sexually dimorphic effects in brain function and structure and in important functioning domains such as mood, learning, and memory. Since smoking addiction is replete with sex differences, future exploration of sex specific, pharmacological interventions targeting specific androgen, or estrogen receptors for prevention or treatment is also warranted.

## Conflict of Interest Statement

The authors declare that the research was conducted in the absence of any commercial or financial relationships that could be construed as a potential conflict of interest.
